# Modulation of intestinal health and metabolism by dietary *Portulaca oleracea* L. extract enhances growth performance, immune function, and meat quality in Wenchang chickens

**DOI:** 10.1016/j.psj.2025.106098

**Published:** 2025-11-12

**Authors:** Yu Zhang, Yaxian Yang, Yinming Li, Yanling Sun, Xiaoyun Han, Hailong Liu, Xinghua Zhao, Yan Zhang, Xin He

**Affiliations:** a College of Veterinary Medicine, Hebei Agricultural University, Baoding, Hebei, 071000, China; b College of Foreign Languages, Hebei Agricultural University, Baoding, Hebei, 071000, China; c lnstitute of Animal Science and Veterinary Medicine, Hainan Academy of Agricultural Sciences, Haikou, 571100, China

**Keywords:** *Portulaca oleracea* L. extract, Wenchang chickens, Meat quality, Gut microbiota, Metabolomics

## Abstract

*Portulaca oleracea* L. extract (POE) shows potential for enhancing poultry production, but its effects on Wenchang chickens are unclear. A total of 90 one-day-old female Wenchang chickens were randomly allocated into three treatment groups: a control group (CON) receiving a basal diet, and two experimental groups (POL and POH) receiving the basal diet supplemented with 0.2 % and 0.4 % POE, respectively. Compared to the CON group, the POL group exhibited significantly higher average daily gain (ADG) and lower feed conversion ratio (FCR) (*P* < 0.05). POL also significantly enhanced immune function: increased serum immunoglobulins (IgG, IgA, IgM), cytokines (IL-1, IL-4, IFN-γ), secretory IgA (sIgA), and spleen index (*P* < 0.05). Meat quality improved in POL, with reduced drip loss, cooking loss, and shear force, alongside increased flavor-enhancing amino acids and inosine monophosphate (IMP) (*P* < 0.05). Jejunal morphology (villus height, VH/CD ratio) and tight junction protein (Claudin-1, Occludin, ZO-1) expression were upregulated in POL (*P* < 0.05). Gut microbiota analysis revealed POL increased *Bacteroides* and *Faecalibacterium* abundance while lowering the Firmicutes / Bacteroidota ratio (*P* < 0.05). Metabolomics indicated enrichment in tryptophan, arginine, and proline metabolism pathways in POL. Dietary 0.2 % POE improved growth, immunity, and meat quality in Wenchang chickens, potentially mediated by enhanced intestinal health and modulation of tryptophan, arginine, and proline metabolism.

## Graphical abstract

**Figure:**
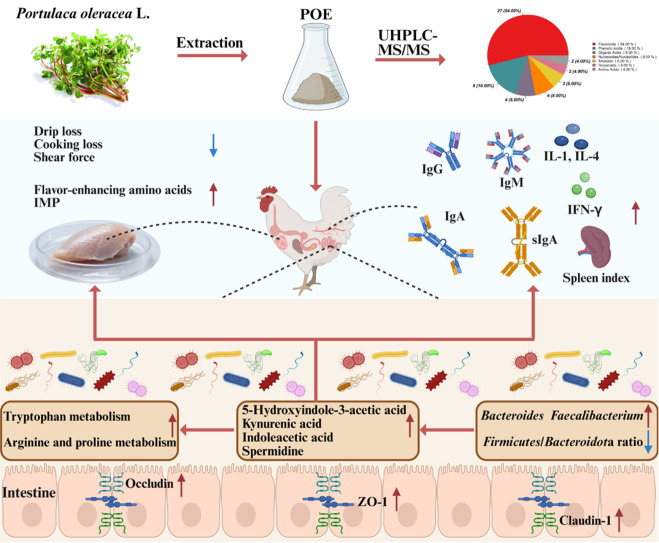

## Introduction

Global antibiotic use in livestock is projected to increase by 11.5 % by 2030, predominantly in low- and middle-income countries (Van Boeckel et al., 2019), raising significant concerns regarding the transmission of antimicrobial resistance through the food chain. In this context, the poultry industry confronts critical challenges: growing consumer demand for antibiotic-free products coexists with increasingly stringent regulatory restrictions on conventional antibiotic-dependent growth promotion practices, due to risks associated with drug residues and the development of resistance (Lone et al., 2021). Recent studies have demonstrated that phytogenic feed additives, which contain natural bioactive compounds, can enhance growth performance, immune function, and intestinal health in poultry, thereby emerging as promising alternatives to antibiotics (Phillips et al., 2023; Aziz-Aliabadi et al., 2024).

*Portulaca oleracea* L., a widely distributed medicinal herb with extensive pharmacological applications, is known to accumulate a variety of bioactive metabolites, including flavonoid glycosides, heteropolysaccharides, and betalain alkaloids. These compounds collectively contribute to its potent reactive oxygen species scavenging capacity, NF-κB-mediated anti-inflammatory activity, and TLR4-dependent immunomodulatory effects (Rahimi et al., 2019; Li et al., 2024). Although the beneficial effects of Portulaca oleracea L. have been reported in quails and rodents (Abd El-Hack et al., 2022; Zhang et al., 2022; Miao et al., 2023; Zhu et al., 2023), there remains a lack of applied research in commercial broiler breeds, particularly in indigenous breeds such as Wenchang chickens. Wenchang chickens, native to Hainan Province in China, are renowned for their superior meat quality and distinctive flavor (Zhang et al., 2023). They represent one of the most economically significant livestock sectors in Hainan Province, generating an output value of USD 1.78 billion in 2020 (Gu et al., 2022).

The gut microbiota plays a crucial role in nutrient metabolism, immune regulation, and the maintenance of intestinal barrier integrity (Caputi et al., 2021; Di Tommaso et al., 2021). Dysbiosis, defined as an imbalance in the composition of microbial communities, has been associated with compromised growth performance, inflammatory responses, and metabolic disturbances in poultry (Xu et al., 2023). Emerging evidence indicates that phytogenic additives can modulate gut microbiota structure and influence host metabolic profiles, thereby contributing to improved productivity (Riva et al., 2024; Wang et al., 2024b; Wu et al., 2024). However, the underlying mechanisms by which *Portulaca oleracea* L. extract (POE) enhances poultry performance—particularly through modulation of the gut microbiome and metabolomic profiles—remain poorly understood. Utilizing 16S rRNA sequencing and untargeted metabolomics analyses, this study provides novel mechanistic insights into the effects of POE in poultry, establishing a scientific basis for its application as a natural growth promoter and intestinal health modulator within sustainable poultry production systems.

## Materials and methods

### Experimental animals and materials

Wenchang chickens were supplied by Qionghai Jinqian Livestock and Poultry Development Co., Ltd. (Hainan, China). Portulaca oleracea L. was sourced from Anguo Chang’an Chinese Medicinal Materials Co., Ltd. (Hebei, China). The plant material was initially identified on the base of morphological characteristics and further authenticated by Prof. Wanyu Shi of Hebei Agricultural University (Hebei, China). A detailed description of the preparation method for POE is provided in the Supplementary Materials.

### Chemical profiling of POE

Ultra-high performance liquid chromatography-tandem mass spectrometry (UHPLC-MS/MS) was employed to identify the major chemical constituents of POE. The detailed analytical methods and associated parameters, including those summarized in Table S1, are described in the Supplementary Materials.

### Experiment design

The animal experiment protocol (No. 2024121) was reviewed and approved by the Animal Protection and Ethics Committee of Hebei Agricultural University. The feeding trial was conducted in the Laboratory Animal Facility of the College of Veterinary Medicine, Hebei Agricultural University. A total of 90 one-day-old female Wenchang chickens were randomly allocated into three treatment groups (five replicates per group, six chickens per replicate): a control (CON) group, a low-dose POE (POL) group, and a high-dose POE (POH) group. Chickens in the CON group received a basal diet, while those in the POL and POH groups were fed the same basal diet supplemented with either 0.2 % (w/w) or 0.4 % (w/w) POE, respectively (Zhao et al., 2013). A photoperiod of 16 h light and 8 h dark was maintained throughout the experimental period, with natural ventilation. The ambient temperature was initially set at 34 °C for the first four days and gradually reduced by 2 °C per week until reaching 24 °C. Relative humidity was maintained at 55-65 %. Routine immunization was conducted using a quadruple live vaccine against Newcastle disease, Infectious bronchitis, Avian influenza H9, and Infectious bursal disease (Lot No. 240131500, Qingdao Yebio Biological Engineering Co., Ltd., Shandong, China). Disinfection protocols included pre-experimental henhouse cleaning with broad-spectrum disinfectants, daily sanitization of feeders and drinkers, and sterility measures for personnel/equipment to prevent cross-contamination. Phase-specific diets (starter: days 1-21; grower: days 22-42), formulated per NRC (1994) and NY/T 33-2004 (Table S2), were adopted in feeding management, with feed supplied twice daily (8:00 a.m. and 5:00 p.m.), ad libitum clean water, and a 12-hour fasting (with access to water) before sampling on days 21 and 42. Regular henhouse inspections monitored chicken health (activity, feed intake, and feces).

### Growth performance

During the experimental period, daily feed intake and residual feed were recorded, and body weight (BW) was measured on days 1, 21, and 42. The average daily gain (ADG), average daily feed intake (ADFI), and feed conversion ratio (FCR) were calculated based on BW, total feed intake, and residual feed. The ADG, ADFI, and FCR were determined in accordance with Eqs. (1), (2), and (3), respectively (Taofeek et al., 2022; Al-Khalaifah et al., 2024; Namted et al., 2025).

(1) $$\text{ADG}\;(g/d)\;=\;\frac{\text{final}\;\text{BW}\;-\;\text{initial}\;\text{BW}}{\text{days}\;\times \;\text{number}\;\text{of}\;\text{chickens}}\;$$

(2) $$\text{ADFI}\;(g/d)\;=\;\frac{\text{overall}\;\text{feed}\;\text{intake}-\text{overall}\;\text{residual}\;\text{feed}}{\text{days}\;\times \;\text{number}\;\text{of}\;\text{chickens}}\;$$

(3) $$\text{FCR}\;=\;\frac{\text{ADFI}}{\text{ADG}}\;$$

### Sample collection

On day 21 of the experiment, chickens were subjected to a 12-hour fasting period prior to blood collection via brachial venipuncture. Serum was separated by centrifugation (4 °C, 3,500 × *g*, 15 min) and stored at −20 °C for subsequent analysis. Following euthanasia by cervical dislocation, lymphoid organs—including the spleen, bursa of Fabricius, and thymus—were excised, weighed, and used to calculate the visceral organ index in accordance with Eq. (4) (Liu et al., 2023). The same fasting protocol was applied on day 42, with additional tissue sampling conducted. Breast muscle samples were collected and divided into aliquots: one portion was flash-frozen in liquid nitrogen for storage at −80 °C, while another was refrigerated at 4 °C for physicochemical analysis. Segments of the jejunum (approximately 1 cm in length) were fixed in 4 % paraformaldehyde solution for histological evaluation. Intestinal mucosa was gently scraped from saline-rinsed jejunal sections using sterile glass slides and stored at −80 °C. Cecal contents were immediately snap-frozen in liquid nitrogen and preserved for subsequent 16S rRNA sequencing and untargeted metabolomic profiling.

(4) $$\text{Viscera}\;\text{index}\;=\;\frac{\text{viscera}\;\text{weight}}{\text{final}\;\text{body}\;\text{weight}}$$

### Serum parameters and intestinal mucosal sIgA concentration

Immunoglobulin G (IgG, JYM0001Ch) kit, immunoglobulin A (IgA, JYM0012Ch) kit, immunoglobulin M (IgM, JYM0060Ch) kit, and secretory immunoglobulin A (sIgA, JYM0036Ch) kit were purchased from Wuhan Colorful Gene Biotech Co., Ltd. (Hubei, China). Interleukin-1 (IL-1, JLC16403) kit was purchased from Jiangxi Jianglan Pure Biological Reagent Co., Ltd. (Jiangxi, China). Interleukin-4 (IL-4, JL21627-48T) kit was purchased from Shanghai Jianglai Biotechnology Co., Ltd. (Shanghai, China). Interferon-γ (IFN-γ, CSB-E08550Ch) kit was purchased from Cusabio Biotech Co., Ltd. (Hubei, China). All measurements were performed at least in triplicate according to the manufacturer's instructions.

### Meat quality

At 45 minutes and 24 hours after excision of breast muscle tissue from the chickens, pH values were measured using a PB-21 pH meter (Sartorius AG, Göttingen, Germany). For quantification of drip loss, 30 g breast muscle samples (designated as W1) were placed in oxygen-permeable polyethylene bags and stored at 4 °C. After 24 h, exudative fluid was removed by blotting with Whatman No. 1 filter paper (11 μm pore size, natural cellulose; Cytiva, UK), and the samples were reweighed (W2). Drip loss was calculated according to Eq. (5) (Zhang et al., 2024). For cooking loss assessment, 30 g samples (W3) were vacuum-sealed in plastic pouches and heated in a water bath maintained at 85 ± 0.5 °C until the internal core temperature reached 75 ± 0.5 °C. Following cooling to ambient temperature (25 ± 0.5 °C), excess surface moisture was removed by blotting, and the samples were weighed (W4). Cooking loss was determined based on Eq. (6) (Zhang et al., 2024). Shear force analysis was conducted on breast muscle strips measuring 1 × 1 × 3 cm, oriented parallel to the myofiber direction, using a C-LM3B texture analyzer (Tenovo International Co., Limited, Beijing, China). Three replicate measurements per sample were averaged for statistical evaluation.

(5) $$\text{Drip}\;\text{loss}\;(\%)\;=\;\frac{W1\;-\;W2}{W1}\;\times \;100\%\;$$

(6) $$\text{Cooking}\;\text{loss}\;(\%)\;=\;\frac{W3\;-\;W4}{W3}\;\times \;100\%\;$$

### Amino acid and inosine monophosphate

To assess meat quality, the concentrations of amino acids and inosine monophosphate (IMP) were quantified in breast muscle tissue samples from Wenchang chickens. Amino acid profiling was performed using an ultra-high performance liquid chromatography system (ACQUITY UPLC I-Class, Waters, MA, USA) coupled with tandem quadrupole mass spectrometry (XEVO TQ-S Micro, Waters, MA, USA). Likewise, IMP content was determined using high-performance liquid chromatography (UPLC I-Class, Waters, MA, USA) with mass spectrometric detection (XEVO TQ-S Micro). Detailed analytical procedures and corresponding instrumental parameters are presented in Tables S3 and S4 of the Supplementary Materials.

### Histological procedures

Jejunum segments were excised from the 4 % paraformaldehyde fixation solution and embedded in paraffin wax. Tissue sections were prepared, stained with hematoxylin and eosin (H&E), and examined using an upright optical microscope (Eclipse E100, Nikon, Japan) equipped with an imaging system (DS-U3, Nikon, Japan). Morphometric analysis of intestinal architecture was performed using microscope image processing software (CaseViewer, Three-Dimensional Histological Technologies, Budapest, Hungary), including measurements of villus height (VH), crypt depth (CD), and the villus height-to-crypt depth ratio (VH/CD).

### RNA extraction and qRT-PCR

Real-time quantitative PCR (RT-qPCR) was employed to assess the expression levels of genes encoding intestinal tight junction proteins, including Claudin-1, Occludin, and ZO-1, in jejunum tissue. Total RNA was extracted from jejunum samples using TRIzol reagent (Vazyme Biotech Co., Ltd., Jiangsu, China). Complementary DNA was subsequently synthesized from the isolated RNA using a T100 Thermal Cycler (Bio-Rad Laboratories, Inc., CA, USA). RT-qPCR amplification was then performed using a 2 × Universal Blue SYBR Green qPCR Master Mix (Wuhan Servicebio Technology Co., Ltd., Hubei, China) on an FQD-96A real-time PCR system (Hangzhou Bioer Technology Co., Ltd., Zhejiang, China). The primer sequences used for amplification are provided in Table S5. Relative mRNA expression levels of the target genes were calculated using the 2^−ΔΔCt^ method, with normalization to the geometric mean of β-actin expression.

### 16S rRNA sequencing

Bacterial DNA was extracted from cecal contents using the cetyltrimethylammonium bromide method, guided by barcode sequence identification. Subsequently, PCR amplification was performed. The primer sequences were specifically designed to distinguish sample data from offline data. The forward primer used was 515F (5′-CCTAYGGGRBGCASCAG-3′), and the reverse primer was 806R (5′-GGACTACNNGGGTATCTAAT-3′), targeting the V4 hypervariable region of the microbial 16S rRNA gene for amplification.

Library construction was carried out using the NEBNext® Ultra™ II FS DNA PCR-Free Library Prep Kit (New England Biolabs (Beijing) Ltd., Beijing, China). Following library preparation, quantification was performed using both Qubit fluorometry and quantitative PCR. Once the library met the required quality standards, paired-end sequencing with a read length of 250 bp was conducted on the NovaSeq 6000 platform (Illumina Corporation, CA, USA).

Raw sequencing reads were assembled using FLASH software (Version 1.2.11) to generate raw tags. Fastp software (Version 0.23.1) was then applied for quality filtering, yielding clean tags. To ensure data accuracy, chimeric sequences were identified and removed by comparing the clean tags against the Silva reference database.

Taxonomic annotation and classification were performed at both the phylum and genus levels using the Silva 138.1 reference database. To assess microbial community diversity, alpha-diversity metrics were calculated using QIIME2 software. For beta-diversity analysis, the unweighted UniFrac distance metric was applied, and the results were visualized through principal coordinate analysis (PCoA) plots. Inter-group differences in species abundance across multiple taxonomic levels were evaluated using independent t-tests. Particular emphasis was placed on taxa exhibiting statistically significant differences. Furthermore, linear discriminant analysis effect size (LEfSe) was conducted using the microeco package, with a linear discriminant analysis (LDA) score threshold of >4.

### Untargeted metabolomics

Cecal tissue samples (100 mg) were individually ground in liquid nitrogen, and the resulting homogenate was resuspended in pre-chilled 80 % methanol by thorough vortex mixing. The samples were incubated on ice for 5 minutes and subsequently centrifuged at 15,000 × *g* at 4 °C for 20 minutes. A portion of the supernatant was diluted to a final methanol concentration of 53 % using LC-MS grade water. The diluted samples were then transferred to fresh Eppendorf tubes and centrifuged under the same conditions (15,000 × *g*, 4 °C, 20 minutes). Finally, the clarified supernatant was collected and injected into the LC-MS/MS system for metabolomic analysis (Want et al., 2012).

UHPLC-MS/MS analyses were conducted using a Vanquish UHPLC system (Thermo Fisher Scientific, MA, USA), coupled with either an Orbitrap Q Exactive HF or Orbitrap Q Exactive HF-X mass spectrometer (Thermo Fisher Scientific, MA, USA). The samples were injected onto a Hypersil Gold column (100 × 2.1 mm, 1.9 μm, Thermo Fisher Scientific, MA, USA), and separated using a 12-minute linear gradient at a flow rate of 0.2 mL/min. Eluent A (0.1 % formic acid in water) and eluent B (methanol) were used for chromatographic separation under both positive and negative ionization modes. The detailed elution gradient profile is provided in Table S6. The Q Exactive HF mass spectrometer was operated in both positive and negative polarity modes, with the following parameters: spray voltage of 3.5 kV, capillary temperature of 320 °C, sheath gas flow rate of 35 psi, auxiliary gas flow rate of 10 L/min, S-lens RF level of 60, and auxiliary gas heater temperature of 350 °C.

### Metabolite identification and pathway analysis

The raw data files generated from the sequencing were imported into the Compound Discoverer 3.3 software platform for metabolite profiling and data processing. Initially, a preliminary screening of key parameters—including retention time and mass-to-charge ratio—was conducted for each detected metabolite. Subsequently, peak area correction was performed using the first quality control (QC) sample to enhance the accuracy of metabolite identification. Peak extraction was then carried out by setting parameters such as a mass deviation threshold of 5 ppm, signal intensity deviation of 30 %, minimum signal intensity, and adduct ion information. During this process, peak areas were also quantified. Following this, target ion integration was executed. Molecular formulas were predicted based on molecular ion peaks and fragment ions, and these were cross-referenced against the mzCloud (https://www.mzcloud.org/), mzVault, and Masslist databases for compound annotation. Background ions were filtered out using blank sample data. The original quantitative results were normalized according to the following formula: Relative peak area = Sample raw quantitative value / ((Sum of sample metabolite quantitative values) / (Sum of QC1 sample metabolite quantitative values)). Metabolites exhibiting a coefficient of variation of relative peak areas exceeding 30 % in QC samples were excluded from further analysis. Finally, the identification and relative quantification results of the remaining metabolites were compiled. Data processing was conducted on a Linux operating system (CentOS release 6.6), utilizing R and Python programming environments. Detailed information regarding specific packages and software versions is provided in the readme file accompanying the results.

Metabolite annotation was performed using the KEGG database (https://www.genome.jp/kegg/pathway.html), HMDB database (https://hmdb.ca/metabolites), and LIPIDMaps database (http://www.lipidmaps.org/). Orthogonal partial least squares discriminant analysis (OPLS-DA) was conducted using the metaX software package (Wen et al., 2017). Univariate statistical analysis, specifically the t-test, was applied to determine the significance of differences between groups based on P-values. Metabolites exhibiting a variable importance in projection (VIP) score > 1, a P-value < 0.05, and a fold change ≥ 2 or ≤ 0.5 were defined as differentially expressed metabolites.Volcano plots were generated using the ggplot2 package in R to visualize significant metabolites based on log2(fold change) and -log10(P-value). For hierarchical clustering heatmaps, the data were normalized by z-score transformation of the intensity values of differential metabolites and visualized using the pheatmap package in R. The correlation among differentially expressed metabolites was assessed using the cor() function in R (Pearson’s method), and the statistical significance of each correlation was calculated using the cor.mtest() function. A P-value threshold of < 0.05 was considered statistically significant. Correlation matrices were visualized using the corrplot package in R. Functional enrichment analysis of the identified metabolites was carried out using the KEGG database. Metabolic pathway enrichment analysis was performed for the differentially expressed metabolites. A metabolic pathway was considered enriched when the ratio x/n exceeded y/N, where x is the number of annotated metabolites in the pathway among the differentially expressed metabolites, n is the total number of annotated metabolites in that pathway, y is the total number of differentially expressed metabolites, and N is the total number of all detected metabolites. Pathways with a P-value < 0.05 were regarded as significantly enriched.

### Statistical analysis

All data were analyzed using SPSS 27.0 software (IBM Inc., NY, USA). The Shapiro-Wilk test and Levene's test were applied to evaluate data normality and homogeneity of variances, respectively. One-way analysis of variance was conducted to assess overall differences among group means, followed by Tukey’s post hoc test for pairwise comparisons. Results were presented as mean ± standard error of the mean, and statistical significance was defined as *P* < 0.05. Data visualization was performed using Origin software (OriginPro 2021, OriginLab Corporation, MA, USA).

## Results

### Chemical profiling of POE

**Fig. 1:**
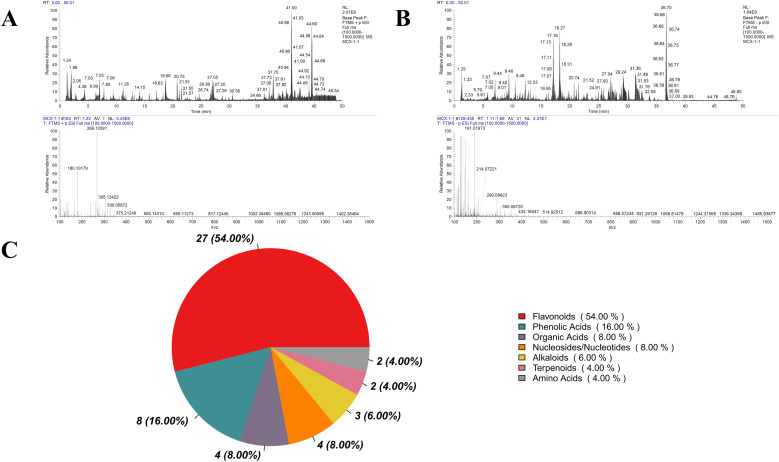
Total ion chromatogram of constituents in POE. (A) positive ion mode. (B) negative ion mode. (C) Pie chart of substance classification.

UHPLC-MS/MS analysis identified a total of 50 distinct chemical compounds in POE. The detection of compounds was dependent on the ionization mode, with 29 compounds detected in positive ion mode and 28 in negative ion mode; among these, 7 compounds were observed in both modes. Total ion chromatograms are displayed in Fig.s 1A and 1B. Based on relative abundance, the identified constituents were classified into different chemical categories (Fig. 1C). Flavonoids represented the most abundant class, accounting for 54 % of all detected compounds, followed by phenolic acids (16 %), organic acids (8 %), nucleosides/nucleotides (8 %), alkaloids (6 %), terpenoids (4 %), and amino acids (4 %). Representative compounds identified include Quercetin, Kaempferol, Phloridzin, Citric acid, and Rutin.

### Effects of POE on growth performance of Wenchang chickens

**Fig. 2:**
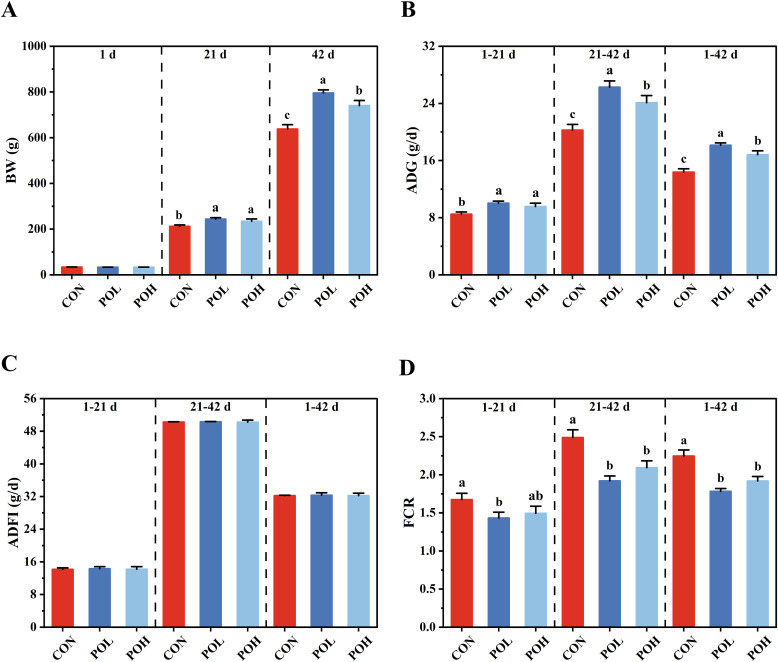
Effects of POE on growth performance of Wenchang chickens. (A) BW at 1 d, 21 d, and 42 d. (B) ADG from 1 d to 21 d, 22 d to 42 d, and 1 d to 42 d. (C) ADFI from 1 d to 21 d, 22 d to 42 d, and 1 d to 42 d. (D) FCR from 1 d to 21 d, 22 d to 42 d, and 1 d to 42 d. Means with different superscripts differ significantly, *P* < 0.05, *n* = 6.

As illustrated in Fig. 2A, the BW of Wenchang chickens at 21 and 42 days of age was significantly higher in the POL and POH groups compared to the CON group (*P* < 0.05). Notably, BW at 42 days of age in the POL group was significantly greater than that in the POH group. In contrast, no significant differences in BW were observed among the CON, POL, and POH groups at 1 d of age (*P* > 0.05). With respect to ADG, values were significantly increased throughout the experimental period in both the POL and POH groups (*P* < 0.05). Moreover, ADG during the periods of 22-42 days and 1-42 days was significantly higher in the POL group than in the POH group. Regarding ADFI, no statistically significant differences were detected among the CON, POL, and POH groups (*P* > 0.05) (Fig. 2C). For FCR, a significant reduction was observed in the POL group during the period of 1-21 days (*P* < 0.05) (Fig. 2D). Further analysis revealed that FCR during the periods of 22-42 days and 1-42 days was significantly improved in both the POL and POH groups (*P* < 0.05).

### Effects of POE on immune function of Wenchang chickens

**Fig. 3:**
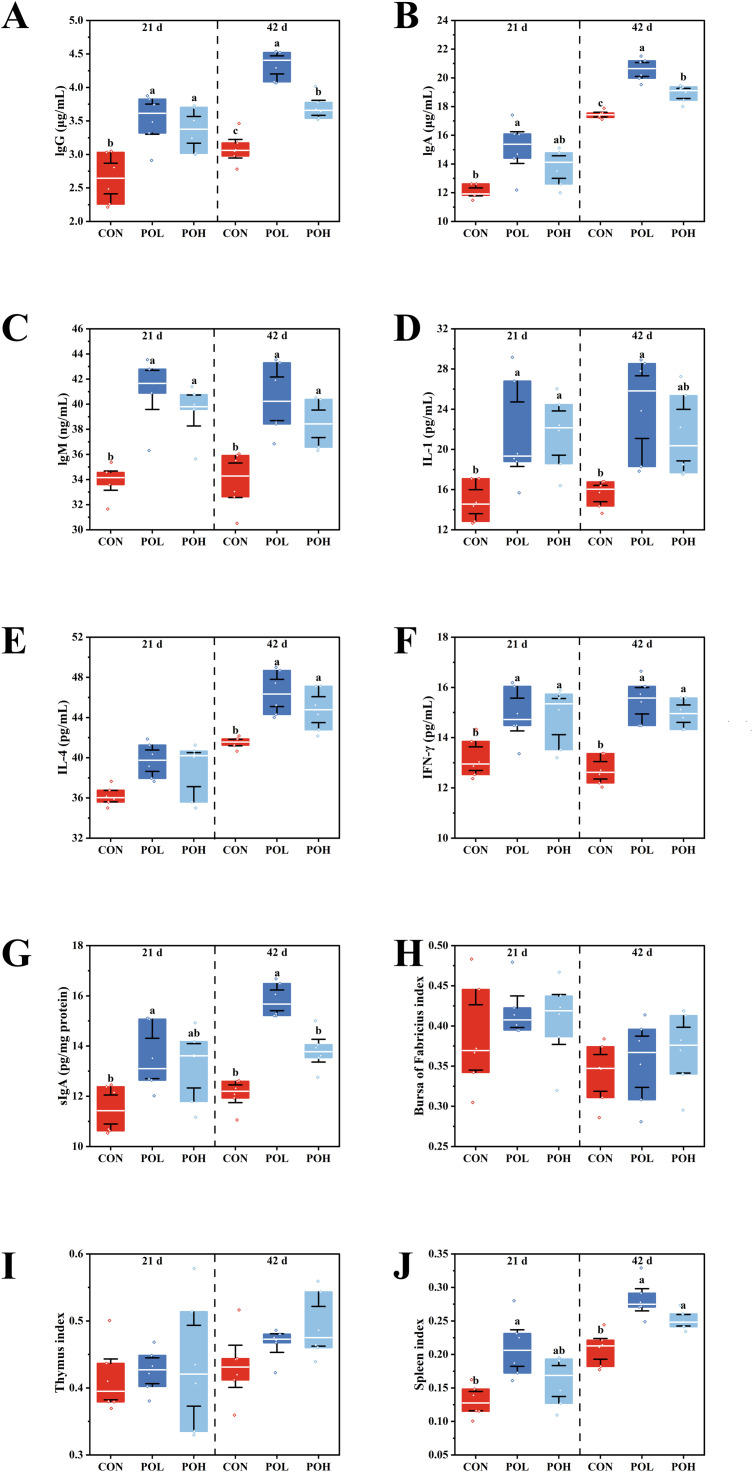
Effects of POE on immune function of Wenchang chickens. (A) IgG at 21 d and 42d. (B) IgA at 21 d and 42d. (C) IgM at 21 d and 42d. (D) IL-1 at 21 d and 42d. (E) IL-4 at 21 d and 42d. (F) IFN-γ at 21 d and 42d. (G) sIgA at 21 d and 42d. (H) Bursa index at 21 d and 42d. (I) Thymus index at 21 d and 42d. (J) Spleen index at 21 d and 42d. Means with different superscripts differ significantly, *P* < 0.05, *n* = 6.

As presented in Fig. 3, the POH group exhibited significantly elevated levels of IgG, IgM, and IFN-γ in Wenchang chickens at both 21 and 42 days of age (*P* < 0.05). The level of IL-1 was significantly increased at 21 days, whereas the levels of IgA, IL-4, and the spleen index were significantly higher at 42 days in the POH group (*P* < 0.05). In the POL group, the concentrations of IgG, IgA, IgM, IL-1, IFN-γ, sIgA, and the spleen index were significantly increased at both 21 and 42 days of age, while the level of IL-4 was significantly elevated only at 42 days (*P* < 0.05). Notably, the POL group demonstrated significantly higher levels of IgG, IgA, and sIgA compared to the POH group at 42 days of age (*P* < 0.05).

### Effects of POE on meat quality of Wenchang chickens

**Fig. 4:**
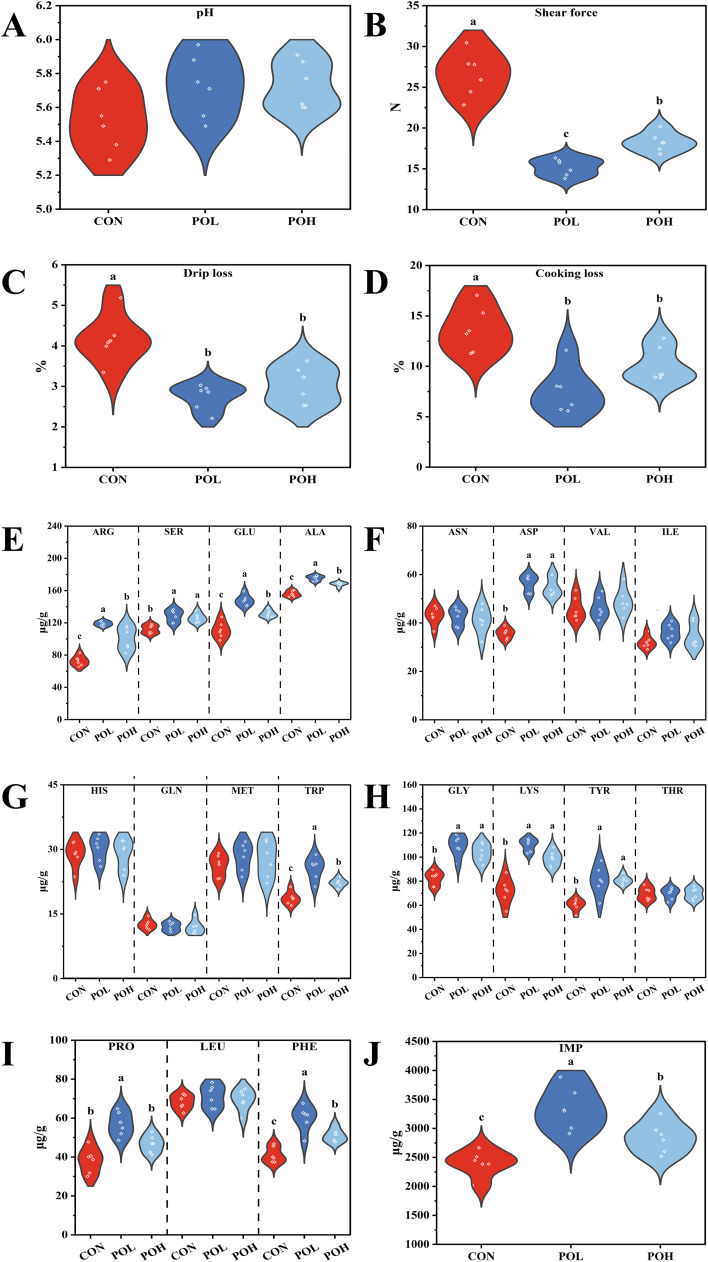
Effects of POE on meat quality of Wenchang chickens. (A) pH. (B) Drip loss. (C) Cooking loss. (D) Shear force. (E) ARG, SER, GLU, and ALA. (F) ASN, ASP, VAL, and ILE. (G) HIS, GLN, MET, and TPR. (H) GLY, LYS, TYR, and THR. (I) PRO, LEU, and PHE. (J) IMP. Means with different superscripts differ significantly, *P* < 0.05, *n* = 6.

Wenchang chickens are well known for their superior meat quality and flavor (Xu et al., 2025). To evaluate the effects of POE on meat quality attributes and umami-related components, analyses of meat quality parameters, amino acid profiles, and IMP content were conducted. As illustrated in Fig. 4, the POH group exhibited significantly reduced drip loss, cooking loss, and shear force, along with elevated concentrations of ARG, SER, GLU, ALA, ASP, TRP, GLY, LYS, TYR, PHE, and IMP (*P* < 0.05). Similarly, the POL group demonstrated decreased drip loss, cooking loss, and shear force, accompanied by increased levels of ARG, SER, GLU, ALA, ASP, TRP, GLY, LYS, TYR, PRO, PHE, and IMP (*P* < 0.05). Furthermore, compared to the POH group, the POL group showed significantly lower shear force and higher concentrations of ARG, GLU, ALA, TRP, PRO, PHE, and IMP (*P* < 0.05).

### Effects of POE on jejunal morphology and tight junction protein expression of Wenchang chickens

**Fig. 5:**
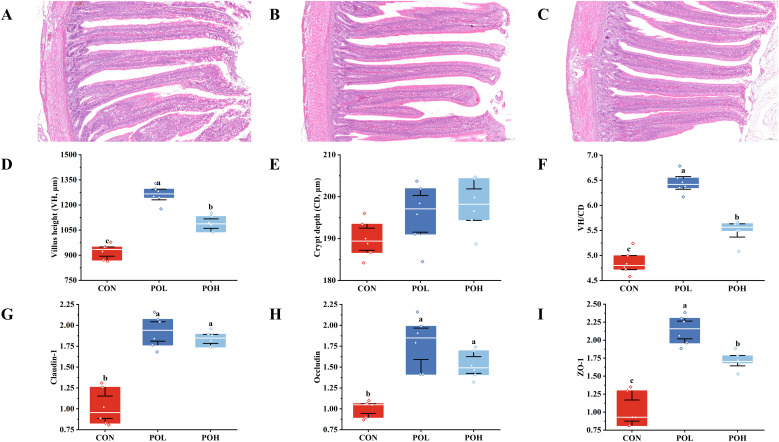
Effects of POE on jejunal morphology and gene expression of Wenchang chickens. (A) Jejunal tissues from the CON group was subjected to H&E staining (100 ×). (B) Jejunal tissues from the POL group was subjected to H&E staining (100 ×). (C) Jejunal tissues from the POH group was subjected to H&E staining (100 ×). (D) VH. (E) CD. (F) VH/CD (G) Claudin-1. (H) Occludin. (I) ZO-1. Means with different superscripts differ significantly, *P* < 0.05, *n* = 6.

As illustrated in Fig.s 5A, 5B, and 5C, the intestinal villi structures were intact and clearly defined across all experimental groups. Notably, the POL and POH groups exhibited more compact, regularly arranged, and morphologically uniform villi compared to the CON group. As presented in Fig.s 5D and 5F, both villus height and the VH/CD ratio were significantly elevated in the POL and POH groups (*P* < 0.05). Specifically, villus height and the VH/CD ratio in the POL group were significantly higher than those observed in the POH group. As shown in Fig. 5E, no statistically significant differences in CD were detected among the three groups (*P* > 0.05). Immunohistochemical analysis (Fig.s 5G, 5H, and 5I) revealed that the expression levels of Claudin-1, Occludin, and ZO-1 were markedly upregulated in both the POL and POH groups (*P* < 0.05). Notably, the POL group exhibited significantly higher expression levels of Claudin-1 and ZO-1 compared to the POH group (*P* < 0.05). These findings indicate that POL exerted a more pronounced beneficial effect on intestinal morphology and barrier function than POH, thereby supporting its selection for subsequent experimental investigations.

### Effects of POE on gut microbiota of Wenchang chickens

**Fig. 6:**
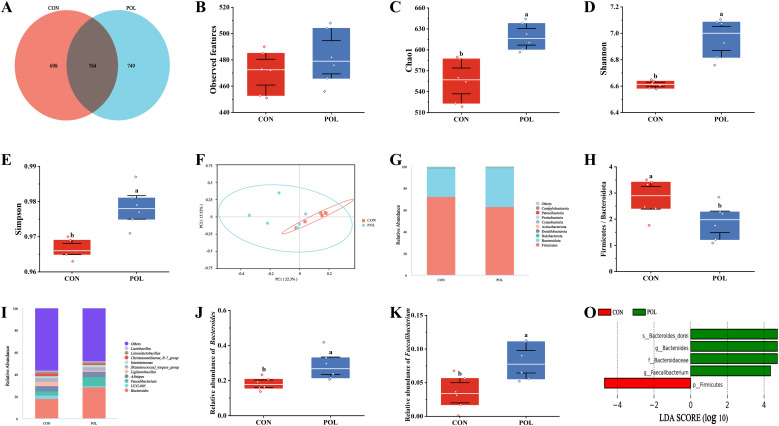
Effects of POE on gut microbiota of Wenchang chickens. (A) The OTUs level Wayne diagram. (B) observed features index (C) chao1 index (D) shannon index. (E) simpson index. (F) PCoA plot based on unweighted unifrac. (G) Relative abundance of gut microbiota at phylum level. (H) Firmicutes / Bacteroidota. (I) Relative abundance of gut microbiota at genus level. (J) Relative abundance of *Bacteroides*. (K) Relative abundance of *Faecalibacterium*. (O) LEfSe analysis. Means with different superscripts differ significantly, *P* < 0.05, *n* = 6.

A total of 2,211 operational taxonomic units (OTUs) were identified through OTU clustering. The CON and POL groups contained 698 and 749 unique OTUs, respectively, with 764 OTUs shared between the two groups (Fig. 6A). The POL group demonstrated significantly higher values for the Chao1 index, Shannon index, and Simpson index compared to the CON group (*P* < 0.05) (Fig.s 6C, 6D, and 6E). However, no statistically significant difference was observed in the number of observed features between the CON and POL groups (*P* > 0.05) (Fig. 6B). PCoA revealed that the microbial community structure of the POL group was significantly distinct from that of the CON group, indicating that supplementation with 0.2 % POE altered the intestinal microbiota composition (Fig. 6F). At the phylum level, Bacteroidota and Firmicutes were the predominant taxa, collectively accounting for over 98 % of the total microbial composition (Fig. 6G). The Firmicutes / Bacteroidota ratio was significantly reduced in the POL group (*P* < 0.05) (Fig. 6H). At the genus level, the dominant genera included *Bacteroides, UCG-005, Faecalibacterium, Alistipes, Ligilactobacillus*, and *[Ruminococcus]_torques_group* (Fig. 6I). Notably, the relative abundances of *Bacteroides* and *Faecalibacterium* were significantly elevated in the POL group (*P* < 0.05) (Fig.s 6J and 6K). Linear discriminant analysis effect size (LEfSe) further confirmed the significant enrichment of s_Bacteroides_dorei, g_Bacteroides, f_Bacteroidaceae, and g_Faecalibacterium in the POL group, identifying them as key differentially abundant taxa. These results underscore the important roles of *Bacteroides* and *Faecalibacterium* in shaping the gut microbiota of the POL group (Fig. 6O).

### Effects of POE on untargeted metabolomics of Wenchang chickens

**Fig. 7:**
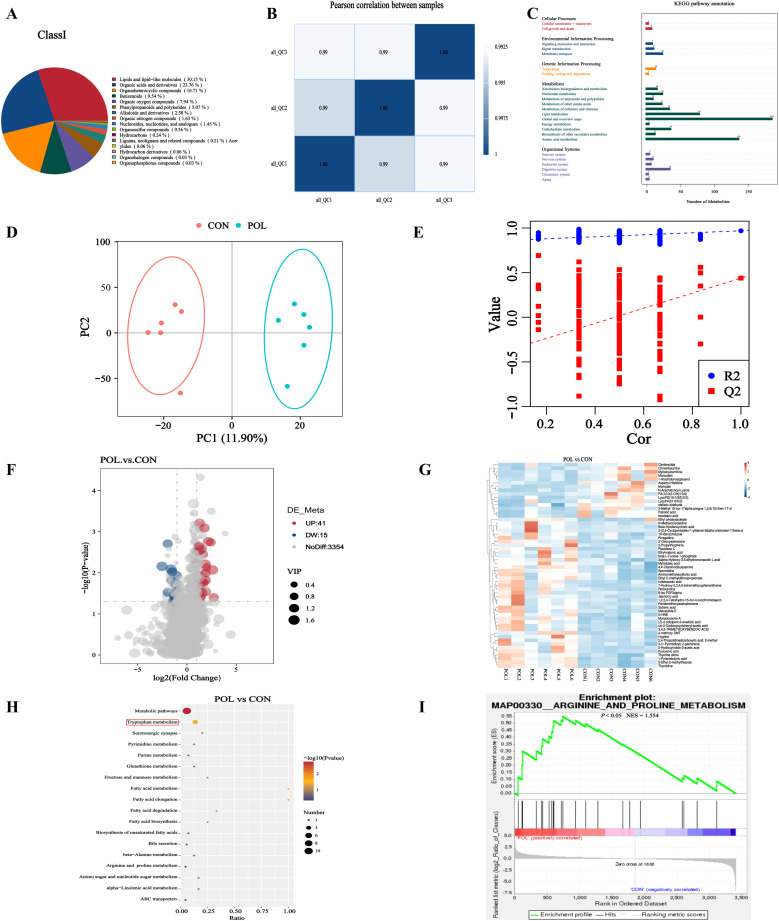
Effects of POE on metabolomics of Wenchang chickens. (A) Pie charts of metabolite Class 1 classification. (B) Correlation of metabolome QC samples (C) KEGG classification annotation of the metabolome. (D) OPLS-DA analysis in POL and CON groups. (E) Permutation test. (F) Volcano plot of the differential metabolites in POL and CON groups. (G) Clustering heat map of differential metabolites in POL and CON groups. (H) KEGG enrichment bubble plot between POL and CON groups. (I) GSEA enrichment plot between POL and CON groups. *n* = 6.

Metabolite classification revealed lipids and lipid-like molecules (30.15 %) as the most abundant class. The subsequent major categories included organic acids and derivatives (23.76 %), organoheterocyclic compounds (16.71 %), benzenoids (9.54 %), and organic oxygen compounds (7.94 %) (Fig. 7A). As illustrated in Fig. 7B, under combined positive and negative ionization modes, the correlation coefficients among QC samples all exceeded 0.99, indicating high reproducibility and reliability of the untargeted metabolomics profiling performed in this study. KEGG pathway analysis demonstrated that the most significantly enriched pathways were associated with global and overview maps, amino acid metabolism, and lipid metabolism (Fig. 7C). OPLS-DA analysis was conducted to compare the metabolite profiles of the CON and 0.2 % POE groups, revealing a clear separation between the two groups (Fig. 7D), which suggests that POE treatment induced significant alterations in the metabolic profile of Wenchang chickens. The validity of the OPLS-DA model was further confirmed by permutation testing (Fig. 7E). Differential metabolite analysis identified a total of 56 metabolites that were significantly altered in the 0.2 % POE group compared to the CON group, including 41 upregulated and 15 downregulated metabolites (Fig. 7F). Hierarchical clustering analysis of these differentially expressed metabolites—defined by VIP > 1, *P* < 0.05, and fold change ≥ 2 or ≤ 0.5—is presented in Fig. 7G. Among the upregulated metabolites were Ethyl pivaloylacetate, N-Methylcorydaldine, Beta-Hyodeoxycholic acid, 3-(2,4-Cyclopentadien-1-ylidene)-5alpha-androstan-17beta-ol, 1H-Benzotriazole, Pivagabine, 2′-Deoxyadenosine, 2-Propylthiophene, Placidene C, Ethylmalonic acid, beta-l-Fucose 1-phosphate, 3alpha-Hydroxy-3,5-dihydromonacolin L acid, Myristoleic acid, 4,4′-Diaminodibutylamine, Spermidine, Aminomethanesulfonic acid, Ethyl 2-(methyldithio)propionate, Indoleacetic acid, 7-Hydroxy-2,3,4,6-tetramethoxyphenanthrene, Rotoxamina, 8-iso PGF3alpha, Jasmonic acid, 1,2,3,4-Tetrahydro-15-nor-4-oxochromolaenin, Pentamethoxyacetophenone, Suberic acid, Mairetolide E, S-HNE, Monascusone A, (Z)-2-octylpent-2-enedioic acid, cis-2-Carboxycyclohexyl-acetic acid, 3,4,5-Trimethoxybenzoic acid, 4‑methoxy DMT, Hygrine, 2,4-Thiazolidinedicarboxylic acid, 2-methyl-, 3-(1-Pyrrolidinyl)-2-pentanone, 5-Hydroxyindole-3-acetic acid, Kynurenic acid, Thymine dimer, 1-Pyrenebutyric acid, 5-Ethyl-2-methylthiazole, Thymidine. Downregulated metabolites included Cardenolide, Chinentaxunine, Myristoylcarnitine, Monoolein, 1-Arachidonoylglycerol, Aspartyl-Histidine, Mompain, N-Arachidonoyl lysine, PA(12:0(3-OH)/10:0), LysoPG(18:1(9Z)/0:0), LysoPA(21:0/0:0), stellatic aldehyde, 3-Methyl-19-nor-17alpha-pregna-1,3,5(10)-trien-17-ol, Palmitic acid, and Isostearic acid. KEGG enrichment analysis indicated that these differentially expressed metabolites were primarily enriched in 17 signaling pathways. Notably, tryptophan metabolism was significantly upregulated following POE administration (*P* < 0.05) (Fig. 7H). Additionally, Gene Set Enrichment Analysis (GSEA) identified the arginine and proline metabolism pathway as the most significantly enriched pathway in response to POE treatment (*P* < 0.05, NES = 1.554) (Fig. 7I).

## Discussion

The POL group demonstrated superior improvements in growth performance, immune function, and meat quality compared to both the POH and CON groups. These findings suggest that moderate POE supplementation enhances nutrient utilization and metabolic efficiency, whereas higher doses do not necessarily yield proportional benefits. A similar phenomenon has been reported in studies involving other herbal extracts, where excessive dosages occasionally resulted in diminished returns (Ghosh et al., 2020; Yan et al., 2022; Guo et al., 2024; El-Abbasy et al., 2025).

Supplementation with POE significantly reduced drip loss, cooking loss, and shear force—three key indicators of meat quality. Lower levels of drip loss and cooking loss contribute to improved juiciness and nutrient retention, while reduced shear force reflects enhanced tenderness, thereby improving meat palatability and consumer acceptability (Cama-Moncunill et al., 2020; Ali et al., 2023). Muscle serves as the primary reservoir of amino acids in the body, and the presence of essential amino acids in meat contributes significantly to its nutritional value (Davis and Fiorotto, 2009; Li et al., 2015). Moreover, the composition of amino acids plays a crucial role in determining meat flavor and represents an important dietary source of essential amino acids for humans (Pereira and Vicente, 2013; Qi et al., 2023). Therefore, the amino acid content in breast muscle tissue was quantified. Our results demonstrated that POE supplementation increased the concentrations of several amino acids, including ARG, SER, GLY, ASP, GLU, ALA, PRO, LYS, TYR, PHE, and TRP. Among these, GLU, ASP, PHE, ALA, GLY, and TYR are collectively classified as flavor-enhancing amino acids, which significantly contribute to umami and overall taste perception (Qi et al., 2023). SER, PRO, and LYS are categorized as sweet-tasting amino acids (Ma et al., 2019). Although TRP has a bitter taste, it plays a regulatory role in RNA, lipid, and protein metabolism in the liver and enhances protein synthesis in both muscle and liver tissues by stimulating insulin secretion (Ma et al., 2019). Furthermore, our study revealed that POE supplementation significantly elevated IMP levels. As a key contributor to umami flavor, the increased IMP content further supports the potential of POE to enhance meat palatability (To et al., 2023). LC-MS analysis confirmed the presence of several bioactive compounds in POE, including Quercetin, Kaempferol, Phloridzin, Citric acid, and Rutin. Importantly, these constituents possess mechanisms relevant to meat quality improvement. Quercetin and Kaempferol inhibit nucleotide-degrading enzymes such as 5′-nucleotidase, thereby prolonging IMP retention and enhancing umami perception (Huang et al., 2020). Phloridzin mitigates gut microbiota dysbiosis by promoting microbial diversity and increasing the production of beneficial metabolites such as short-chain fatty acids, IMP, and d-(−)-beta-hydroxybutyric acid (BHB) (Zhang et al., 2025). Citric acid improves water-holding capacity and tenderness while simultaneously suppressing lipid oxidation (Ke et al., 2009). Rutin enhances lipid profiles by increasing intramuscular fat and n-3 polyunsaturated fatty acid (PUFA) content, resulting in improved antioxidant activity, reduced drip loss, and a more favorable n-6/n-3 PUFA ratio (Li et al., 2023). Collectively, these findings provide strong evidence supporting the hypothesis that dietary supplementation with POE enhances meat quality in Wenchang chickens.

The gut microbiota plays a crucial role in nutrient absorption, immune system development, and disease resistance through the production of vitamins, short-chain fatty acids (SCFAs), organic acids, and antimicrobial compounds (Shang et al., 2018). Gut bacteria and their metabolites have also been shown to influence skeletal muscle metabolism and function via the “gut-muscle axis” (Grosicki et al., 2017; Frampton et al., 2020). In chickens, the composition and diversity of the gut microbiota are closely associated with production performance (Siegerstetter et al., 2017). Modulation of the gut microbiota through supplementation with probiotics from genera such as *Lactobacillus, Bacillus, Saccharomyces*, and *Citrobacter* has demonstrated beneficial effects on BW and FCR in both chickens and other animal species (Feng et al., 2022). Moreover, accumulating evidence supports the positive impact of probiotic bacteria on chicken meat quality, which encompasses traits such as meat color, pH, texture, and water-holding capacity (Maltin et al., 2003). For example, *Enterococcus faecium, Streptococcus faecium*, and *Bacillus subtilis* have all been reported to enhance meat quality. These findings provide foundational evidence that gut microbiota can influence chicken muscle physiology and metabolism (Zheng et al., 2014; Wang et al., 2017; Cramer et al., 2018; Trabelsi et al., 2019). Microbial analysis revealed that dietary supplementation with 0.2 % POE increased the abundance of beneficial bacterial genera, including *Bacteroides* and *Faecalibacterium*, while reducing the Firmicutes / Bacteroidota ratio. Bacteroides is known to be involved in the production of short-chain fatty acids, particularly acetate (Fan et al., 2023). Enhanced SCFAs production by gut microbes has been linked to improved meat quality attributes, such as meat color, marbling score, water-holding capacity, and the accumulation of beneficial fatty acids in muscle tissue (Niu et al., 2022). Acetate promotes skeletal muscle growth, development, and metabolic activity by upregulating Gm16062 expression (Frampton et al., 2020; Kobayashi et al., 2024; Yang et al., 2024). Increased acetate intake has also been associated with elevated glycogen content in pig muscle, which serves as a primary energy reserve for muscle growth (Imoto and Namioka, 1983). A recent study demonstrated that acetate supplementation in germ-free mice resulted in increased body mass, enhanced succinate dehydrogenase activity, and upregulated myogenesis-related gene expression in skeletal muscle (Yang et al., 2024), indicating that acetate plays a central role in regulating muscle development and mediating the gut microbiota-muscle axis. *Faecalibacterium* contributes to butyrate production through the fermentation of indigestible fibers—another major SCFA that serves as a key energy source for intestinal epithelial cells and has been shown to promote muscle development via mitochondrial biogenesis (Yamamoto et al., 2022). An elevated Firmicutes / Bacteroidota ratio has been associated with impaired host amino acid metabolism, including disruptions in lysine degradation, arginine biosynthesis, and phenylalanine metabolism (Jiang et al., 2022). Collectively, these findings indicate that POE enhances meat quality in Wenchang chickens through the coordinated regulation of gut microbiota.

The gut microbiota reshapes the host amino acid landscape through efficient metabolism of intestinal amino acids (Li et al., 2024). Upon transport to muscle tissues via systemic circulation, these amino acids can directly modulate muscle function and metabolism (Lahiri et al., 2019). In this study, advanced metabolomic profiling techniques were employed to comprehensively characterize the metabolic profiles of Wenchang chickens. The results clearly identified tryptophan metabolism as the predominant metabolic pathway. Tryptophan metabolism encompasses three major routes: the kynurenine pathway, the 5-hydroxytryptamine pathway, and the indole pathway (Xue et al., 2023). Kynurenine undergoes catabolism within muscle tissues, and its downstream metabolism has been shown to alleviate stress responses in both the intestine and central nervous system (Cervenka et al., 2017). 5-Hydroxytryptamine influences endocrine and metabolic signaling pathways in muscle cells, thereby promoting cellular proliferation and tissue development (Field et al., 2021). The indole pathway, mediated by gut microbiota, converts tryptophan into indole and its derivatives, which contribute to intestinal homeostasis by regulating the expression of pro-inflammatory and anti-inflammatory cytokines (Miyamoto et al., 2024). More importantly, GSEA revealed that POE significantly enhanced arginine and proline metabolism in Wenchang chickens, with a notable upregulation of spermidine. Arginine is efficiently absorbed by the intestinal epithelium, enters systemic circulation, and reaches peripheral tissues such as breast muscle, where it stimulates protein synthesis and cell proliferation (Zampiga et al., 2018). Additionally, arginine enhances the utilization of ketone bodies in peripheral tissues, including skeletal muscle, where they are oxidized to generate energy (Zampiga et al., 2018). Proline is converted to arginine via the intermediate pyrroline-5-carboxylate (Wang et al., 2024a), and the arginine-proline axis constitutes a central regulatory node in amino acid metabolism (Wang et al., 2024a). This axis also serves as a metabolic scaffold for the synthesis of other nonessential amino acids and essential metabolites (Kuo et al., 2021). Proline catabolism yields glutamate, which enters the tricarboxylic acid (TCA) cycle to produce ATP, thereby ensuring sustained energy supply (Wang et al., 2024a). Furthermore, proline metabolism generates reducing equivalents that enter the mitochondrial electron transport chain to drive ATP synthesis (Phang et al., 2010). Alternatively, proline degradation can yield α-ketoglutarate, an intermediate of the TCA cycle (Zampiga et al., 2018). Spermidine has been demonstrated to exert significant effects on multiple cellular processes, including regulation of cell proliferation and differentiation, protein synthesis, physical activity, muscle development, antioxidant defense, and stress response mechanisms (Pegg, 2016). Collectively, the present findings indicate that dietary supplementation with POE improves meat quality in Wenchang chickens by modulating key amino acid metabolic pathways, particularly those involving tryptophan, arginine, and proline. These insights not only enhance our understanding of the metabolic mechanisms underlying POE’s biological effects but also provide a scientific foundation for the rational use of POE as a functional feed additive to improve poultry growth performance, immune function, and meat quality. Future studies should aim to elucidate the specific molecular mechanisms linking these metabolic pathways, including the roles of key enzymes (e.g., arginase, proline dehydrogenase), transcriptional regulators, and their interactions with gut microbiota and host genetic factors.

The study was restricted to a single poultry breed, gender, and growth stage: we only used one-day-old female Wenchang chickens and evaluated effects over a 42-day period (covering starter and grower phases). In commercial poultry production, farmers often raise multiple indigenous or hybrid breeds, and both male and female chickens are reared for different purposes (e.g., males for meat production, females for egg-laying in some systems). Additionally, Wenchang chickens may have a longer production cycle (e.g., up to 120 days for optimal meat flavor) in practical farming, and our short-term (42-day) observation cannot fully reflect the long-term effects of POE supplementation—such as whether its benefits persist into the finishing phase, or if there are potential cumulative impacts on muscle development or metabolic homeostasis. Thus, the generalizability of our results to other breeds, genders, or longer growth cycles remains to be verified.

## Conclusions

Dietary supplementation with POE enhanced growth performance, immune function, and meat quality in Wenchang chickens through the modulation of intestinal health and the regulation of tryptophan, arginine, and proline metabolism. These findings offer valuable insights for future research aimed at optimizing the use of natural growth promoters in poultry production. However, further investigations are necessary to elucidate the precise molecular mechanisms underlying POE-mediated regulation of gut microbiota and metabolic pathways.

## Ethical approval

The animal study was approved by the Institutional Animal Care and Use Committee of Hebei Agricultural University. The study was conducted in accordance with the local legislation and institutional requirements.

## Funding

This work was supported by the 10.13039/501100001809National Natural Science Foundation of China (No. 32473073, 32172898) and the Institute-level Scientific Research Project of the Institute of Animal Husbandry and Veterinary Medicine, Hainan Academy of Agricultural Sciences (HNXM2024ZD03).

**Availability of data and materials:** The datasets used during the current study are available from the corresponding author on reasonable request. The datasets of intestinal flora sequencing generated during the current study are available in the NCBI repository, National Center for Biotechnology Information (nih.gov), with the bioproject number PRJNA1290854.

## CRediT authorship contribution statement

**Yu Zhang:** Writing – review & editing, Writing – original draft, Formal analysis, Data curation. **Yaxian Yang:** Validation, Investigation. **Yinming Li:** Writing – review & editing. **Yanling Sun:** Validation, Investigation. **Xiaoyun Han:** Writing – review & editing, Formal analysis, Data curation. **Hailong Liu:** Writing – review & editing, Project administration. **Xinghua Zhao:** Writing – review & editing, Funding acquisition. **Yan Zhang:** Writing – review & editing, Funding acquisition, Conceptualization. **Xin He:** Writing – review & editing, Supervision, Project administration, Funding acquisition.

## Disclosures

The authors declare that they have no conflicts of interest.

## Appendix Supplementary materials

Image, application 1

Image, application 2

## Footnotes

Supplementary material associated with this article can be found, in the online version, at doi:10.1016/j.psj.2025.106098.
