# Supplementary material for: Modulation of intestinal health and metabolism by dietary Portulaca oleracea L. extract enhances growth performance, immune function, and meat quality in Wenchang chickens
Source: Poult Sci. 2025 Nov 12;105(1):106098. doi: 10.1016/j.psj.2025.106098 (PMC12666711; doi:10.1016/j.psj.2025.106098)
Supplement: Supplementary file 1 [file mmc1.docx]

Modulation of Intestinal Health and Metabolism by Dietary *Portulaca oleracea* L. Extract Enhances Growth Performance, Immune Function, and Meat Quality in Wenchang Chickens

Yu Zhang^a^, Yaxian Yang^a^, Yanling Sun^a^, Xiaoyun Han^a^, Hailong Liu^b^, Xinghua Zhao^a*^, Yan Zhang^b*^, Xin He^a*^

^a^ College of Veterinary Medicine, Hebei Agricultural University, Baoding, Hebei, 071000, China.

^b^ lnstitute of Animal Science and Veterinary Medicine, Hainan Academy of Agricultural Sciences, Haikou, 571100, China

* Corresponding author:

Xinghua Zhao, Address: No.2596, Lekai South Street, Baoding, 071000, China

E-mail: [xianzhaoxinghua@163.com](mailto:xianzhaoxinghua@163.com)

Yan Zhang, Address: No.14, Xingdan Road, Haikou, 571100, China

E-mail: zy79818_0@163.com

Xin He, Address: No.2596, Lekai South Street, Baoding, 071000, China

E-mail: dyhexin@hebau.edu.cn

Supplementary materials

Preparation method of *Portulaca oleracea* L. extract

200 g of *Portulaca oleracea* L. were soaked in water at a ratio of 1:10 (w/v) for 1 h, followed by reflux extraction for 1 h to get the first decoction (DLSB-10 Low-Temperature Coolant Circulating Pump, Beijing Jinfurenhao Technology Development Co., Ltd., Beijing, China) (DHT Stirring temperature regulating electric heating sleeve, Shandong Juancheng Hualu Electric Heating Instrument Co., Ltd., Shandong, China). In the second extraction, 8 times the volume of water (relative to the mass of *Portulaca oleracea* L.) was employed, and the extraction time was 1 h. The decoction was combined and the relative density was 1.1, and then dried under reduced pressure at 60 °C (DZF-6123 Vacuum drying oven, Shanghai One Instrument Science Instrument Co., Ltd., Shanghai, China). After the extraction process, 46.62 g of *Portulaca oleracea* L. extract was obtained. Therefore, the amount of *Portulaca oleracea* L. corresponding to 1 g of *Portulaca oleracea* L. extract (POE) was 4.29 g.

Chemical profiling of POE

0.1 g of POE was weighed and 10 mL of 70% methanol solution was added. Ultrasonic-assisted extraction was carried out (for 30 min at room temperature), followed by centrifugation (at 10,000 rpm for 10 min), and the supernatant was collected. The extraction procedure was repeated once, and the supernatants were combined. The combined solution was dried under a stream of nitrogen. The residue was redissolved in 0.5 mL of methanol, filtered through a 0.2 μm membrane filter, and then subjected to instrumental analysis.

UHPLC-MS/MS analyses were performed using a Thermo Scientific Vanquish Flex ultra-high performance liquid chromatography system (Thermo Fisher Scientific, MA, USA) coupled with an Orbitrap Exploris 120 mass spectrometry system (Thermo Fisher Scientific, MA, USA). Samples were injected onto a Hypersil Gold column (100×2.1 mm, 1.9μm) using a 50-min linear gradient at a flow rate of 0.4 mL/min. The eluents for the positive and negative polarity modes were eluent A (0.1% FA in ACN) and eluent B (0.1% FA in H₂O). The elution gradient is shown in Table S1. Orbitrap Exploris 120 mass spectrometer was operated in positive/negative polarity mode with spray voltages of 3.5 kV (+) and 3.2 kV (-), capillary temperature of 320 ℃, sheath gas flow rate of 45 psi and aux gas flow rate of 15 L/min, S-lens RF level of 70, Aux gas heater temperature of 350 ℃.

Data were collected using TraceFinder 5.2 (Thermo Fisher Scientific, MA, USA). Characteristic peaks were extracted and background noise was removed using Thermo Xcalibur Qual Explorer (Thermo Fisher Scientific, MA, USA). TraceFinder 5.2 was then used to match the exact mass number (with an error ≤ 5 ppm), isotopic distribution (> 70%), and the number of product ions (≥ 1, with an error ≤ 5) for comparison with the database.

Amino acid in breast meat tissue

Sample preparation: An appropriate amount (approximately 50 mg) of the sample was stored at 4 ℃, thawed, homogenized, and then precisely weighed. Subsequently, 1000 μL of 50% methanol/water was added, and the mixture was extracted with shaking for 1 h. The centrifuge tube was placed in a cryogenic centrifuge and centrifuged at 12,000 rpm for 10 min at 4 ℃. A total of 10 μL of the supernatant was transferred into a 1.5 mL centrifuge tube, and then 10 μL of ultrapure water, 5 μL of an internal standard, and 40 μL of isopropanol (containing 0.1% formic acid) were added. The mixture was vortexed and shaken for 2 min. The centrifuge tube was again placed in a cryogenic centrifuge and centrifuged at 12,000 rpm for 10 min at 4 ℃. Next, 10 μL of the supernatant was taken and placed in a 1.5 mL centrifuge tube, 70 μL of borate buffer was added, followed by the addition of 20 μL of accQ Tag derivatization reagent (Kairos amino acid kit, Waters, MA, USA). The supernatant was immediately shaken for 10 seconds. After 1 min, the excess derivative was hydrolyzed, and the derivatization reaction was terminated. The centrifuge tube was heated at 55 ℃ for 10 min. Finally, 400 μL of water was added for dilution prior to measurement.

Preparation of standards: The standard substance was prepared using the gradient dilution method. The concentration of each amino acid solution was adjusted to 400, 200, 100, 40, 20, 10, 4, 2, and 1 μmol/L. Firstly, 20 μL of the sample, 5 μL of the internal standard, and 40 μL of isopropanol (containing 0.1% formic acid) were vortexed and shaken for 2 min. The centrifuge tube was then placed in a cryogenic centrifuge and centrifuged at 12,000 rpm for 10 min at 4 ℃. Subsequently, 10 μL of the supernatant was transferred into a 1.5 mL centrifuge tube, 70 μL of borate buffer was added, and 20 μL of accQ Tag derivatization reagent (Kairos amino acid kit, Waters, MA, USA) was added. The supernatant was immediately shaken for 10 seconds. After 1 min, the excess derivative was hydrolyzed, and the derivatization reaction was terminated. The centrifuge tube was heated at 55 ℃ for 10 min. Then, 400 μL of water was added for dilution. Finally, 100 μL of the supernatant was transferred into a liquid-phase vial for testing.

Samples were separated on a Waters UPLC HSS T3 (1.8 μm, 2.1 mm × 150 mm, Waters, MA, USA) using a Waters ACQUITY UPLC I-CLASS ultra-high performance liquid chromatography system (Waters, MA, USA), at a flow rate of 0.5 mL/min. Mobile phase A was 0.1% formic acid aqueous solution, and mobile phase B was acetonitrile. The elution gradient is shown in Table S3. The injection volume was 5 μL, and the column temperature was 50 ℃. Mass spectrometry was performed using a Waters XEVO TQ-S Micro tandem quadrupole mass spectrometry system (Waters, MA, USA). The positive ion source voltage was 1.5 kV, the cone voltage was 20 V, the desolvation temperature was 600 ℃, the desolvation gas flow rate was 1000 L/h, and the gas flow rate of the cone hole was 10 L/h.

The peak area was calculated using the MassLynx quantitative software (Waters, MA, USA), and the allowable error for the retention time was 15 s. The quantitative results were obtained through the standard curve method.

Inosine monophosphate in breast meat tissue

Samples were taken, placed at 4 ℃, thawed, and then approximately 50 mg of each sample was weighed and homogenized. 1 mL of 80% methanol was added, and the mixture was homogenized. The centrifuge tube was placed in a cryogenic centrifuge and centrifuged at 12000 rpm for 10 min at 4 ℃. The supernatant was removed, diluted 50-fold with ultrapure water, and transferred to liquid-phase vials for mass spectrometry. The content of IMP in breast meat tissue was determined using high performance liquid chromatography (UPLC I-Class, Waters, MA, USA).

The separation was performed on Waters UPLC BEH C8 (1.7 μm, 2.1 mm × 100 mm, Waters, MA, USA) at a flow rate of 0.3 mL/min. Mobile phase A was 0.5% ammonium acetate aqueous solution, and mobile phase B was acetonitrile. The elution gradient is shown in Table S4. The column temperature was 45 ℃. Mass spectrometry was performed using a Waters XEVO TQ-S Micro tandem quadrupole mass spectrometry system (Waters, MA, USA). The positive ion source voltage was 3 kV, and the cone voltage was 20 V. The ion source temperature was 150 ℃, and the desolvation temperature was 450 ℃. The desolvation gas flow rate was 950 L/h, and the gas flow rate of the cone hole was 10 L/h.

The peak area of the target data was calculated using the TargetLynx quantitative software (Waters, MA, USA), with an allowable retention time error of 15 s. The concentration was calculated using the standard curve method to obtain the quantitative results.

Table S1. Elution gradient of POE

| Time (min) | Mobile phase | |
| --- | --- | --- |
|  | A (v%) | B (v%) |
| 0 | 5 | 95 |
| 2 | 5 | 95 |
| 42 | 95 | 5 |
| 46.9 | 95 | 5 |
| 47 | 5 | 95 |
| 50 | 5 | 95 |

Table S2. Composition and nutrient levels of the standard diet (dry matter basis, %).

| Item | 1~21 d | 22~42 d |
| --- | --- | --- |
| Maize | 52.50 | 58.80 |
| Soybean meal | 40.00 | 33.80 |
| Soybean oil | 3.00 | 3.00 |
| Dicalcium phosphate | 1.9 | 1.8 |
| Limestone powder | 1.08 | 1.22 |
| Salt | 0.37 | 0.37 |
| Lysine | 0.05 | 0.03 |
| Methionine | 0.19 | 0.07 |
| Premix^1^ | 0.80 | 0.80 |
| Choline chloride | 0.11 | 0.11 |
| Total | 100.00 | 100.00 |
| Nutritional levels^2^ |  |  |
| Metabolisable energy (MJ/kg) | 12.42 | 12.62 |
| Crude protein | 21.77 | 19.65 |
| Calcium | 1.00 | 1.02 |
| Available phosphorus | 0.44 | 0.42 |
| Lysine | 1.34 | 1.15 |
| Methionine | 0.55 | 0.40 |
| Cystine | 0.40 | 0.36 |

^1^Premix provided the following per kilogram of diet: vitamin A 9000 IU; vitamin D_3_ 3000 IU; vitamin E 26 IU; vitamin K_3_ 1.20 mg; vitamin B_1_ 3.00 mg; vitamin B_2_ 8.00 mg; vitamin B_6_ 4.40 mg; vitamin B_12_ 0.012 mg; niacin 45 mg; calcium pantothenate 15 mg; folic acid 0.75 mg; biotin 0.20 mg; choline chloride 1100 mg; Fe 100 mg; Cu 10 mg; Zn 108 mg; Mn 120 mg; I 1.5 mg; and Se 0.35 mg. ^2^Crude protein was the measured value, while the others were all calculated values.

Table S3. Elution gradient of amino acids.

| Time (min) | Mobile phase | |
| --- | --- | --- |
|  | A (v%) | B (v%) |
| 0 | 96 | 4 |
| 0.5 | 96 | 4 |
| 2.5 | 90 | 10 |
| 5 | 72 | 28 |
| 6 | 5 | 95 |
| 7 | 5 | 95 |
| 7.1 | 96 | 4 |
| 9 | 96 | 4 |

Table S4. Elution gradient of inosine monophosphate.

| Time (min) | Mobile phase | |
| --- | --- | --- |
|  | A (v%) | B (v%) |
| 0 | 95 | 5 |
| 0.5 | 95 | 5 |
| 2 | 15 | 85 |
| 3 | 0 | 100 |
| 4 | 0 | 100 |
| 4.1 | 95 | 5 |
| 6 | 95 | 5 |

Table S5 The primer sequences for qRT-PCR

| Gene name | Primer sequence (5´→3´) | Accession number | Product size, bp |
| --- | --- | --- | --- |
| *β-actin* | Forward:  TGCGTGACATCAAGGAGAAG  Reverse: TGCCAGGGTACATTGTGGTA | NM_205518.2 | 300 |
| *Claudin-1* | Forward: GTCTTTGGTGGCGTGATCTT  Reverse: TCTGGTGTTAACGGGTGTGA | NM_001013611.2 | 117 |
| *Occludin* | Forward: GCTGAGATGGACAGCATCAA  Reverse: CCTCTGCCACATCCTGGTAT | NM_046904540.1 | 97 |
| *ZO-1* | Forward: TGGCAATCAACTTTGGGTAGCA  Reverse: ATCCACAGAGGCAACTGAACCATA | NM_046925214.1 | 156 |

Table S6. Elution gradient of untargeted metabolomics analysis

| Time (min) | Mobile phase | |
| --- | --- | --- |
|  | A (v%) | B (v%) |
| 0 | 98 | 2 |
| 1.5 | 98 | 2 |
| 3 | 15 | 85 |
| 10 | 0 | 100 |
| 10.1 | 98 | 2 |
| 11 | 98 | 2 |
| 12 | 98 | 2 |
